# Speech-Stimulating Substances in Autism Spectrum Disorders

**DOI:** 10.3390/bs9060060

**Published:** 2019-06-12

**Authors:** María Andrea Castillo, Kendy Eduardo Urdaneta, Neomar Semprún-Hernández, Anna Lisa Brigida, Nicola Antonucci, Stephen Schultz, Dario Siniscalco

**Affiliations:** 1 Research Division, Autism Immunology Unit of Maracaibo, Maracaibo 4001, Venezuela; mandicb0@gmail.com (M.A.C.); kurdanetag@gmail.com (K.E.U.); neomar.semprun@gmail.com (N.S.-H.); 2 Department of Biology, Faculty of Sciences, University of Zulia, Maracaibo 4001, Venezuela; 3 Catedra libre de Autismo, Universidad del Zulia, Maracaibo 4001, Venezuela; 4 Italian Group for Studying Autism—GISA, 25018 Brescia, Italy; brigida.annalisa@gmail.com; 5 Biomedical Centre for Autism Research and Treatment, 70126 Bari, Italy; info@antonucci.eu; 6 Department of Cellular and Integrative Physiology, School of Medicine, University of Texas Health Science Center San Antonio, San Antonio, TX 78229, USA; stevendri0629@gmail.com; 7 Department of Experimental Medicine, University of Campania, 80138 Napoli, Italy; 8 Centre for Autism-La Forza del Silenzio, 81036 Caserta, Italy

**Keywords:** autism spectrum disorder, speech, language, nutrition

## Abstract

Autism spectrum disorder (ASD) is characterized by the core domains of persistent deficits in social communication and restricted-repetitive patterns of behaviors, interests, or activities. A heterogeneous and complex set of neurodevelopmental conditions are grouped in the spectrum. Pro-inflammatory events and immune system dysfunctions are cellular and molecular events associated with ASD. Several conditions co-occur with ASD: seizures, gastro-intestinal problems, attention deficit, anxiety and depression, and sleep problems. However, language and speech issues are key components of ASD symptoms current therapies find difficult to face. Several speech-stimulating substances have been shown to be effective in increasing speech ability in ASD subjects. The need for large clinical trials to determine safety and efficacy is recommended.

## 1. Biological Aspects of Speech and Verbal Communication in Autism Spectrum Disorder (ASD)

Autism Spectrum Disorder (ASD) is defined by the Diagnostic and Statistical Manual for Mental Disorders, Fifth Edition (DSM-5), with one of the prominent features being persistent deficits in social communication. These symptoms begin in early childhood, and produce clinically significant deficits in the social use of verbal and non-verbal communication [1]. The latest edition of the DSM, DSM-5, combined the previously separate subtypes of ASD listed in DSM-4. Autistic disorder, Asperger syndrome, pervasive developmental disorder-not otherwise specified (PDD-NOS), and Childhood Disintegrative Disorder are now combined into one diagnosis of ASD with these categories indicating varying levels of severity and age of onset along the autism spectrum [1,2].

Some features of ASD, also commonly called autism, are seen in genetic and chromosomal abnormalities such as fragile X syndrome, Down Syndrome, as well as in many identified genomic insertions and deletions; however, most cases of ASD have an unknown etiology which indicates they could be due to environmental factors. Two of the prominent clinical features of ASD are inflammation and neuro-immune system dysregulation [3,4,5]. The US Centers for Disease Control and Prevention (CDC) estimates that ASD occurs in one of every 59 children in the US aged eight years old [6], while an estimate from Xu and colleagues (2018) using data from the National Health Interview Survey puts the estimate of ASD higher when including children 3–17 years of age, where they found one child affected out of every 40 children in the US for the years 2014–2016 [7]. In a 2013 review article, we have summarized environmental factors which could contribute to ASD pathogenesis through epigenetic modifications [8]. Since the publication of that review, additional articles have continued to add to the evidence of epigenetic modifications in ASD. Some of these epigenetic modifications include DNA methylation, epigenetic proteins, gene polymorphisms associated with variation in diet, histone modifications, and microRNA dysregulation [9,10,11,12].

Some parents report regression in their children or a loss of previously acquired verbal skills with the subsequent diagnosis of ASD [13]. Parental reports of regression in children with ASD is estimated to occur in approximately 22% of cases [14]. Parental reports of regression have been validated with the use of videotape of children’s first and second birthdays [15]. Since these children did not initially present with symptoms of ASD, their verbal regression may be due to environmental factors to which a child is exposed, such as nutrition and medication use.

## 2. Speech-Stimulating Substances in ASD: Overview

Many substances have been proposed to improve speech in individuals with ASD. Vitamins in particular have been proposed as therapies. Vitamin B6 has been well-studied as a possible therapy after the Autism Research Institute in the US found that many parents saw improvements in their children with high Vitamin B6 doses [16]. Vitamin B12 has been much investigated showing its involvement in ASD [17]. Vitamin D has been suggested as a therapy to improve symptoms of ASD including speech [18]. Although various vitamins have shown positive results in some children, no vitamin has shown effectiveness in all children with ASD. Contrarily, a study published in 2018 by Bittker and Bell showed a weak positive association between Vitamin D drops and increased risk of ASD [19]. This study also showed increased risk for ASD from acetaminophen use and decreased use of breastfeeding as we have also seen [20,21].

Arachidonic acid (ARA), a polyunsaturated omega-6 fatty acid, may improve the speech of children with ASD. Arachidonic acid (ARA) is considered a conditionally essential nutrient in infants which is present in breast milk but not all infant formulas [20]. Although infants can produce ARA, they do not produce as much as is required for their development and must acquire some from their diets [22]. ARA is required for production of the endocannabinoids anandamide and 2-arachidonylglycerol (2-AG). A study of piglets showed that arachidonic acid and other essential fatty acids in the diet affect the levels of anandamide and other endocannabinoids in the brain [23]. Anandamide and 2-AG are the primary signaling molecules in the endocannabinoid system (ECS) [24]. Anandamide is the primary ligand for cannabinoid receptor 1 (CB1) which is primarily found in the brain and is responsible for regulating neurite outgrowth in the brain as well as for synapse positioning [25]. 2-AG is the primary ligand for CB2 receptors which are primarily found on immune system cells and regulates their function [26]. A deficiency of ARA could lead to lower levels of anandamide and 2-AG, which could be the mechanism for the increased ASD risk we have shown due to a lack of sufficient amounts of breastfeeding or use of an infant formula without ARA supplementation [20].

We have recently shown that the atypical cannabinoid palmitoylethanolamide (PEA) improved speech in a report of two cases of ASD [27]. Messenger RNA (mRNA) for the production of CB2 receptors is up-regulated in the peripheral blood mononuclear cells (PBMCs) of individuals with ASD as we have shown [28]. This up-regulation of receptors could be the result of insufficient endocannabinoids in the blood. Our paper from 2008 shows an association of acetaminophen use with increased risk for ASD [21]. In this paper, reported use of acetaminophen at age 12–18 months significantly increased the odds of a child having ASD by more than eight times. Acetaminophen produces analgesia by indirectly stimulating CB1 receptors [29], which we suggested could produce dysregulation of the ECS to produce ASD [30]. Recently, it has been shown that anandamide levels are low in the blood of individuals with ASD [31], which supports our hypothesis of ECS dysregulation in ASD.

The following paragraphs will analyze the speech-stimulating substances methylcobalamin, tetrahydrobiopterin, folinic acid, omega-3 polyunsaturated fatty acids, flavonoids, and other medications with ASD in greater detail.

### 2.1. Methylcobalamin (Vitamin B12)

Methylcobalamin,(IUPAC:cobalt(3+);[(2~{R},3~{S},4~{R},5~{S})-5-(5,6-dimethylbenzimidazol-1-yl)-4-hydroxy-2-(hydroxymethyl)oxolan-3-yl]1-[3[(1~{R},2~{R},3~{R},5~{Z},7~{S},10~{Z},12~{S},13~{S},15~{Z},17~{S},18~{S},19~{R})-2,13,18-tris(2-amino-2-oxoethyl)-7,12,17-tris(3-amino-3-oxopropyl)-3,5,8,8,13,15,18,19-octamethyl-2,7,12,17-tetrahydro-1~{H}-corrin-24-id-3-yl]propanoylamino]propan-2-yl phosphate, mecobalamin, MeCbl, or MeB12) is the active form of cobalamin, also known as vitamin B12 [32]. It is a cofactor of the methionine synthase enzyme, which catalyzes the transfer of methyl groups [33]. Methylcobalamin is actively taken up by neurons, and it has been indicated for the treatment of nervous disorders through effective systemic or local delivery [32]. Its use in treating autism has been proposed as a complementary treatment [34]. Restoration of the impaired methylation capacity in children with ASD with the use of methylcobalamin, together with folinic acid and betaine, was demonstrated early [35]. However, vitamin B12 injected (64.5 µg/kg every three days, subcutaneously) in a 12-week, double-blind, placebo-controlled, cross-over clinical trial of 30 children with ASD showed no effect on overall outcomes [36]. Of note, a subset of treated children improved both behavioral and oxidative stress measures, indicating an active role of methyl B12 in reducing oxidative stress [36]. No speech analysis was performed in this study. A larger open-label trial with the use of 75 µg/Kg methylcobalamin, twice daily, together with folinic acid, demonstrated improvement in autistic symptoms, glutathione redox status and expressive communication. Receptive, expressive, and written language showed marked improvements [37]. These beneficial effects could be due to the re-balance in glutathione redox status and, thus, in redox metabolism [38].

### 2.2. Tetrahydrobiopterin (THB)

Tetrahydrobiopterin (THB) (IUPAC:(6~{R})-2-amino-6-[(1~{R},2~{S})-1,2-dihydroxypropyl]-5,6,7,8-tetrahydro-1~{H}-pteridin-4-one, BH4, sapropterin) is a cofactor of the three aromatic amino acids, phenylalanine, tryptophan and tyrosine, hydroxylase enzymes [39]. These enzymes catalyze the hydroxylation of their respective substrates. Mutations in the genes encoding for these enzymes could be responsible for neurocognitive, neuropsychiatric, and developmental problems, as THB is required for the synthesis of several neurotransmitters [40]. An early study demonstrated that THB was reduced in the cerebrospinal fluid of children with ASD with respect to controls [41]. Using chromatographic techniques, the authors demonstrated that the brain of autistic subjects showed a dysregulated endogenous biosynthesis of THB. Following that research study, it was proposed that ASD could be a consequence of limited cofactor availability [42], and it was suggested that THB could be helpful in reducing autistic symptoms. Oral administration (1 mg/kg per day) of 6R-L-erythro-5,6,7,8-tetrahydrobiopterin (R-THBP) in 14 autistic children was partially effective in improving autistic behavior, as seven children displayed improvements and seven children were non-responders [43]. A small group of autistic children was further treated for three months with THB [44]. The subjects reported improvements in social functioning, eye contact and interaction, as well as increased R-THB levels in cerebrospinal fluid. Interestingly and for the first time, speech improved after THB administration, as the number of words or sounds increased [44]. More recently, lower concentrations of THB were found in the spinal cords of autistic subjects [45]. The children were further treated with 3 mg/Kg per day of THB for six months alternating with placebo. After six months of treatment, they showed a significant improvement in the social interaction score [45]. In 2010, Frye et al. reviewed the clinical trials performed on the use of THB in autism [46]. Summarizing all the results, marked improvements were seen in cognitive ability, social interaction, communication, and verbal capacity. Side effects were not noted; however, a definitive, standard protocol needs to be defined to harmonize dose, time of treatment, and outcomes. Later, the same author performed an open-label clinical trial with the use of 20 mg/Kg per day of THB in 10 autistic children for 16 weeks [47]. Most notably, marked improvements in the Preschool Language Scale were seen. Nitric oxide metabolism parameters were also changed. Moreover, THB could be a critical element for the synthesis of the precursors of monoamine neurotransmitters, such as dopamine and norepinephrine, and is vital in nitric oxide production [48]. It has been proposed that chronic environmental gestational exposure to nitrous oxide could impact ASD development [48].

A larger (46 Children with ASD) double-blind, placebo-controlled trial with the use of 20 mg/kg/day THB or placebo for 16 weeks demonstrated the effectiveness in reducing problems with social awareness, autism mannerisms, hyperactivity, and inappropriate speech [49]. A reanalysis of three clinical trials [50] has shown that THB does improve concomitant metabolic abnormalities in individuals with ASD; in particular, it has a significant effect on methylation and markers of chronic oxidative stress; however, additional clinical trials would be required to conclusively establish a beneficial effect on speech.

### 2.3. Folinic Acid

Folinic acid (IUPAC:2-[[4-[(2-amino-5-formyl-4-oxo-3,6,7,8-tetrahydropteridin-6-yl)methylamino]benzoyl]amino]pentanedioic acid, formyltetrahydrofolate, leucovorin) is a 5-formyl derivative of tetrahydrofolic acid which has similar effects to folic acid. Maternal folic acid and multivitamin supplementation before and during pregnancy are now well recognized as nutritional treatments for reducing ASD risk [51]. It has already been demonstrated that the use of folinic acid together with methylcobalamin as nutritional intervention is effective in reducing redox imbalances in autistic children [38]. It has been proposed that a subgroup of children with ASD, bearing the presence of folate receptor autoantibodies, would benefit of treatment with leucovorin calcium [52]. Indeed, four months of treatment has been able to improve verbal communication, receptive and expressive language. Folate receptor autoantibodies are able to block folate uptake, disrupting its pathway [53], causing cerebral folate deficiency [54]. This syndrome has been associated with autism [55]. A subgroup of children with ASD with these autoantibodies could represent as a particular ASD subset (but with high incidence, as at least 70% of autistic children show positivity for these autoantibodies [56]) of patients that could respond to high doses of folinic acid [57]. As stated in paragraph 2.1, synergistic treatment with methylcobalamin (subcutaneous injection) and folinic acid (400 μg as powder mixed in food, twice a day, orally) showed efficacy in ameliorating speech problems in children with ASD [37]. Consequently, a large double-blind placebo-controlled trial was initiated to determine if high doses of folinic acid were efficacious in improving verbal communication and language impairments [58]. Forty-eight autistic children received 2  mg/ kg per day, maximum 50  mg per day, of folinic acid for 12 weeks. Folinic acid improved verbal communication with respect to placebo; more importantly, greatest improvements in speech were seen in treated autistic children with high folate receptor autoantibodies [58]. As an explanation, folinic acid can easily cross the blood–brain barrier by using the reduced folate carrier when the folate receptors are blocked or dysfunctional. In addition, folinic acid does not require catalytic reduction by the enzyme dihydrofolate reductase and can readily enter the folate cycle to be used as a metabolite [58].

### 2.4. Omega-3 Polyunsaturated Fatty Acids

The term omega-3 polyunsaturated fatty acids (PUFA) indicates a group of carboxylic acids with three C-C double bounds (IUPAC: (4~{Z},7~{Z},10~{Z},13~{Z},16~{Z},19~{Z})-docosa-4,7,10,13,16,19-hexaenoic acid;(5~{Z},8~{Z},11~{Z},14~{Z},17~{Z})-icosa-5,8,11,14,17-pentaenoic acid;(9~{Z},12~{Z},15~{Z})-octadeca-9,12,15-trienoic acid). PUFAs are essential components of cellular membranes, and they are required by external intake from diet, such as from fish oil, as these biomolecules cannot be endogenously synthesized in the body.

In a typically developing population, it has been demonstrated that higher maternal fish intake during pregnancy is associated with higher language and communication skills, assessed in 15/18-month children [59]. Furthermore, lower levels of maternal seafood consumption in pregnancy has been associated with suboptimal levels of social and language development [60]. Maternal fish intake (more than twice weekly servings), compared with never consumed, has been directly associated with higher cognitive and language development at age three years [61]. However, to avoid the presence of potential neurotoxic substances, fish should be cleaned from environmental pollutants. Complementary supplementation of omega(ω)-3 in ASD is still an open research debate [62], as many trials have achieved conflicting results. In the valproic acid (VPA)-induced autism animal model, it has been demonstrated that there is a neuro-protective effect mediated by ω-3/-6 acids [63] that possesses immunomodulatory and anti-inflammatory capabilities [64]. Furthermore, blood levels of ω-3 fatty acids are decreased in children with ASD [65]. Whereas ω-3 fatty acid supplementation (eight weeks) improved autistic behaviors in a randomized, crossover, placebo-controlled study [66], as well as improved hyperactivity, lethargy, and stereotypy in children with ASD [67], ω-3/-6/-9fatty acids were effective in ameliorating language abilities in preterm children with ASD [68]. In this randomized double-blind placebo-controlled clinical trial, the authors demonstrated that three months of oral treatment with ω-3/-6/-9 fatty acids were able to increase the number of words produced, the combined gesture and word use, and the broader social communicative gesture in 18–38 months, preterm born, ASD toddlers. They concluded that supplementation with PUFAs positively affected overall social communication [68]. Interestingly, possible explanation of these positive effects of PUFAs arose from an animal study demonstrating that dietary ω-3 polyunsaturated fatty acid supplementation was able to restore alterations in the expression of several genes [69]. Most recently, a randomized, large (n = 73), placebo-controlled trial demonstrated the efficacy of combined treatment with vitamin D and ω-3 PUFAs in increasing social communicative functions in children with ASD [70]. In general, ω-3 PUFAs demonstrated beneficial effects on metabolic function and in reducing inflammation [71].

### 2.5. Flavonoids (Luteolin) and Corticosteroids (Prednisolone and Prednisone)

The flavonoids-derived compound mixture (luteolin, quercetin and rutin) Neuroprotek® has a high gut absorption because of its olive kernel oil formulation, and many studies have assessed its properties. A trial was performed with ASD individuals (37 pediatric subjects) who were enrolled in a four-month regime with an administration of at least 2 capsules/20 kg weight, equivalent to at least 400 mg total flavonoid (each capsule contains 200mg of flavonoids). An important outcome was observed by the authors, which was the resumption of speech in 10% of the children [72]. A chemical feature particularly of luteolin is the capacity to cross the blood-brain barrier [73] and to exert its biological effects in the central nervous system. The resumption of speech may be due to luteolin’s antioxidant (reducing brain oxidative stress), anti-inflammatory (reducing gut and brain inflammation), anti-allergy (inhibiting mast cells and microglia) and neuroprotective properties. It also stabilizes blood vessels and promotes neuronal recovery. This compound mixture of flavonoids is safe [74], well-tolerated and may ameliorate ASD symptoms with an improvement in verbal language capabilities [72].

Corticosteroids exert their effect by affecting gene transcription and translation and modulating enzyme activity. The clinical roles of corticosteroids are related to their anti-inflammatory effects and immune-modulating properties due to their inhibitory effects on phospholipase A2, an enzyme required for the production of inflammatory compounds [75].

A few authors have studied corticosteroid therapy in the treatment of neurodevelopmental diseases with language improvements noted after treatment. A two-case clinical trial of children diagnosed with Childhood Disintegrative Disorder (CDD) was performed [76]. The first case was treated with prednisolone (2 mg/kg/day) and started speaking again by day 11, and afterwards showed normal academic school progress at a 30-month follow-up. The second case was also treated with prednisolone (2 mg/kg for two weeks, tapered over one week), and over the next month she started developing a little speech; improvement continued at 48-month follow-up [76].

The corticosteroid specific pathway that could explain how these molecules improve verbal language remains unclear, although most neurodevelopmental disorders, including ASD, have physiological, metabolic, and neuropsychologically similar disturbances among them [77]. In an early clinical case, the authors studied a child with language and behavior regression at 22 months diagnosed with pervasive developmental disorder. An empirical course of corticosteroid treatment was initiated after initial evaluation at six years of age when a 28-week trial of corticosteroid (starting with 2mg/kg of prednisone) was begun. Over several weeks of treatment, there was a significant increase in spontaneous speech and greater improvement in verbal communication skills. Language regression may be due to a neuropathogenic alteration and corticosteroid response may be optimal in early onset of the disorder, but without amelioration of nonverbal cognitive abilities. However, corticosteroid therapy could be a possible therapy for speech resumption in neurodevelopmental disorders such as ASD.

### 2.6. Other Medications (Alzheimer’s Medications and Beta-Adrenergic Antagonism (Propranolol))

ASD treatments are highly individualized, and include biomedical drug treatments, special education, and targeted speech therapy. People with ASD have bloodstream hyperserotonemia in contrast to decreased levels of serotonin in the central nervous system [78]. Some study cases have reported the usage of Alzheimer’s medications to treat ASD symptoms especially those related to language and speech. Hertzman (2003) reported three clinical cases of autistic adults treated with galantamine (4 mg daily at bedtime). All of them, within the first month started active speech, production of sounds, responding appropriately, and had improved cognition [79]. It had a positive effect on verbalization, socially appropriate behavior or both, in two cases; however, many side effects were reported in the second case. Speech improvement was not observed in the third case although some other beneficial effects were seen, such as diminished aggressive behavior [79].

Galantamine is a cholinomimetic compound; its dual effects combine competitive inhibition of acetylcholinesterase and allosteric modulation of nicotinic receptors. This produces increased acetylcholine in the neural synapse, while also increasing allosterically sodium and calcium influx. This leads to the release of glutamate, dopamine, and serotonin into the synaptic cleft [80]. Despite the positive effects observed in the treatment of autism, biochemical pathways need further research in order to decrease chances for harm. 

In another report, novel Alzheimer drugs have been assessed in ASD treatment, including galantamine, rivastigmine, tacrine, and memantine; these studies reported improvements in receptive language, social interaction expressive language and communication [81], positioning these medications as promising drugs to future treatment of ASD.

Noradrenergic receptors play a role in a broad range of brain functions such as arousal, stress response, memory consolidation, sleep/wakefulness, learning, signal detection, and more [82,83,84]. Noradrenergic activity has been suggested to be increased in the ASD population [85,86]. Autistic features include problems in utilization of contextual information and processing semantic information [87,88]. Mehler and Purpura (2009) proposed a developmental dysregulation of the noradrenergic system in ASD individuals, relating it to improved behaviors and enhanced communication seen sometimes during fever in these individuals [89]. Indeed, they postulated that ASD core behaviors are the result of the developmental dysregulation of the noradrenergic system and neural network deployment.

Propranolol is a ß1,2-adrenoreceptor antagonist clinically used to target peripheral sites of the noradrenergic system and can be deployed to block ß-adrenoreceptors in the central nervous system, since as a lipophilic compound it readily crosses the blood–brain barrier [90]. It appears to affect flexibility of access to lexical, semantic and associative networks on verbal problem-solving tasks in neurotypical individuals [87]. Propranolol has been tested within the ASD population in clinical assays, showing cognitive benefits on verbal problem solving without significant effects on anxiety levels [91,92]. In an open trial with eight autistic adults, subjects received propranolol for a period of 11–19 months [93]; four of them improved their speech and another patient learned to point. Authors believe that these effects are a result of a decrease in the autistic individual’s state of hyperarousal. Recently, a single-dose trial reported an improvement in performance on conversational reciprocity without modifications of anxiety levels [94]. Even though there are few studies on propranolol as a supplement for ASD and speech, and their sample sizes are very small, the existing evidence suggests that propranolol could be a pharmacological agent to diminish these processing network problems and have cognitive benefits.

## 3. Future Perspectives of a Protocol to Stimulate Verbal Communication in ASD

We have correlated acetaminophen use at the time of childhood vaccination with the prevalence of ASD [21] and have proposed that the endocannabinoid system of children with ASD could have been dysregulated from acetaminophen use [95]. We have shown that children with ASD have more mRNA for type 2 cannabinoid receptors in their PBMCs compared to the typically-developing population [28]. In a further study, we showed that acetaminophen use for fever is associated with ASD and hypothesized that children with ASD would have lower endocannabinoid tone after frequent over-activation of the endocannabinoid system from acetaminophen use [30]. Recent studies have shown that this is correct: children with ASD have lower levels of three key cannabinoids in their blood: anandamide (N-arachidonoylethanolamine or AEA), N-palmitoylethanolamine (PEA), and N-oleoylethanolamine (OEA) [96]. PEA and OEA can be orally administered to increase blood levels of these endocannabinoids; however, anandamide is rapidly metabolized in the body and can be increased only indirectly. Administration of CBD of up to 600 mg/day in adults has been shown to increase blood levels of anandamide, perhaps by competitive inhibition of fatty acid amide hydrolase (FAAH), the enzyme responsible for anandamide degradation [97].

We have previously shown the beneficial effects PEA administration in two subjects [26]. Administration of CBD and THC to individuals with ASD has been shown to be safe and efficacious as well as to improve ASD symptoms in a recent parental survey [98]. We propose a clinical trial to study the effects of orally administered CBD, PEA, and OEA to raise the blood levels of anandamide, PEA, and OEA to normal levels in individuals with ASD. We believe our study would have the most desirable effects on speech by including OEA and PEA in addition to CBD in the treatment regimen. Further, we expect that normalizing the blood levels of OEA, PEA, and anandamide by this treatment will have beneficial effects on behavior in individuals with ASD.

## 4. Conclusions

**Figure 1:**
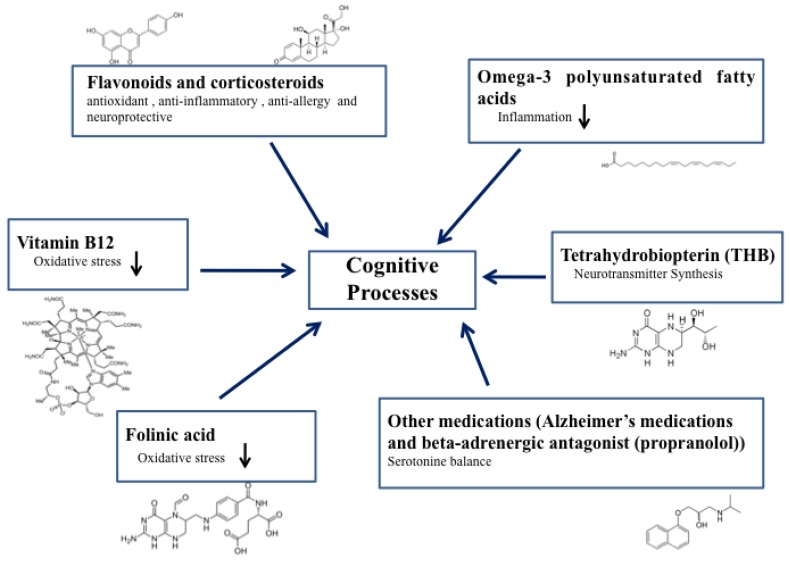
Key elements of cognitive process regulation through substances discussed in the manuscript and their chemical structures.

This review considers all the substances that have been proposed to improve core ASD features, specifically those related with the speech (Figure 1). Before claiming enthusiastic outcomes, it is noteworthy to consider the need of large clinical trials to determine the speech-stimulating substances efficacy and safety. However, remarkable results are based on case reports, small size samples and often open-label studies. These studies show that the efficacy of speech-stimulating substances in ASD appears to be encouraging. As of today, scientist and clinicians have enough knowledge about vitamin B6, arachidonic acid, methylcobalamin, tetrahydrobiopterin, folinic acid, omega-3 polyunsaturated fatty acids, luteolin, prednisolone, prednisone, propranolol and Alzheimer drugs (galantamine, rivastigmine, tacrine, memantine).

The use of these substances is also encouraged by their low rate of side effects. In addition, all these vitamins, lipids, steroids, beta blockers, Alzheimer drugs and other metabolites have an oral route of administration with safe dosage ranges. Knowing the pharmacological mechanisms of action of these substances, we hypothesize that immunological alterations are involved in the lack of speech pathogenesis. Hence, through ameliorating the dysregulated inflammatory responses, a better treatment for ASD core features could be addressed. Therefore, translational medicine studies should be performed using these substances in order to establish a safe new protocol to treat ASD-mediated lack of speech ability.
